# Genome-Guided Mass Spectrometry Expedited the Discovery of Paraplantaricin TC318, a Lantibiotic Produced by *Lactobacillus paraplantarum* Strain Isolated From Cheese

**DOI:** 10.3389/fmicb.2020.01381

**Published:** 2020-07-14

**Authors:** Walaa E. Hussein, En Huang, Ismet Ozturk, Árpád Somogyi, Xu Yang, Baosheng Liu, Ahmed E. Yousef

**Affiliations:** ^1^Department of Food Science and Technology, The Ohio State University, Columbus, OH, United States; ^2^Department of Microbiology and Immunology, National Research Center, Giza, Egypt; ^3^Department of Environmental and Occupational Health, University of Arkansas for Medical Sciences, Little Rock, AR, United States; ^4^Faculty of Fine Arts, Department of Gastronomy and Culinary Arts, Istanbul Arel University, Istanbul, Turkey; ^5^Campus Chemical Instrument Center, Mass Spectrometry and Proteomics Facility, The Ohio State University, Columbus, OH, United States; ^6^Nutrition and Food Science Department, California State Polytechnic University Pomona, Pomona, CA, United States; ^7^College of Animal Science Technology, Jiangxi Agricultural University, Nanchang, China; ^8^Department of Microbiology, The Ohio State University, Columbus, OH, United States

**Keywords:** bacteriocins, lantibiotics, genome-guided MS, *Lactobacillus paraplantarum*, paraplantaricin

## Abstract

The quest for potent alternatives to the currently used antimicrobials is urged by health professionals, considering the rapid rise in resistance to preservatives and antibiotics among pathogens. The current study was initiated to search for novel and effective bacteriocins from food microbes, preferably lactic acid bacteria (LAB), for potential use as preservatives. Advances in genome-guided mass spectrometry (MS) were implemented to expedite identifying and elucidating the structure of the recovered antimicrobial agent. A LAB strain, OSY-TC318, was isolated from a Turkish cheese, and the crude extract of the cultured strain inhibited the growth of various pathogenic and spoilage bacteria such as *Bacillus cereus*, *Clostridium sporogenes*, *Enterococcus faecalis*, *Listeria monocytogenes*, *Salmonella enterica* ser. Typhimurium, and *Staphylococcus aureus*. The antimicrobial producer was identified as *Lactobacillus paraplantarum* using MS biotyping and genomic analysis. Additionally, *L. paraplantarum* OSY-TC318 was distinguished from closely related strains using comparative genomic analysis. Based on *in silico* analysis, the genome of the new strain contained a complete lantibiotic biosynthetic gene cluster, encoding a novel lantibiotic that was designated as paraplantaricin TC318. The bioinformatic analysis of the gene cluster led to the prediction of the biosynthetic pathway, amino acid sequence, and theoretical molecular mass of paraplantaricin TC318. To verify the genomic analysis predictions, paraplantaricin TC318 was purified from the producer cellular crude extract using liquid chromatography, followed by structural elucidation using Fourier transform ion cyclotron resonance MS analysis. This genome-guided MS analysis revealed that the molecular mass of paraplantaricin TC318 is 2,263.900 Da, its chemical formula is C_106_H_133_N_27_O_22_S_4_, and its primary sequence is F-K-S-W-S-L-C-T-F-G-C-G-H-T-G-S-F-N-S-F-C-C. This lantibiotic, which differs from mutacin 1140 at positions 9, 12, 13, and 20, is considered a new member of the epidermin group in class I lantibiotics. In conclusion, the study revealed a new *L. paraplantarum* strain producing a novel lantibiotic that is potentially useful in food and medical applications.

## Introduction

Antimicrobial agents are used in food and medical industries to control hazardous microorganisms. The risk of exposure to these microorganisms is increasing, while their resistance to conventionally used antimicrobials is also on the rise (Verraes et al., 2013; Sharma et al., 2018). Additionally, consumers are increasingly avoiding foods containing synthetic antimicrobials and prefer products with clean labels (Doyle et al., 2015). Driven by these urgencies, investigators are prompted to search for novel, potent, and natural antimicrobials to replace the synthetic ones and combat the emerging resistant microorganisms. Bacteriocins are natural, ribosomally synthesized, occasionally posttranslationally modified antimicrobial peptides produced by bacteria (Riley and Wertz, 2002). Among the bacteriocin-producing bacteria, lactic acid bacteria (LAB) are a desirable source of these antimicrobials (Stiles and Holzapfel, 1997). Lactic acid bacteria have been used safely for decades to manufacture fermented foods due to their role in flavor and texture development (Abee, 1995; Stiles, 1996). LAB bacteriocins are potentially promising as a safe and effective alternative to synthetic food preservatives and antibiotics (Cleveland et al., 2001; Cotter et al., 2013; Cavera et al., 2015).

Lactic acid bacteria bacteriocins have been grouped into three classes: (I) <5-kDa, lanthionine-containing peptides, often referred to as lantibiotics; (II) <10-kDa non-lanthionine-containing peptides; and (III) large bacteriolysins (Perez et al., 2014). The lantibiotics group has been classified further, according to the biosynthetic pathway, into four classes (Willey and van der Donk, 2007; van der Donk and Nair, 2014). Class I lantibiotics are modified by LanB (dehydratase) and LanC (cyclase) enzymes. Class II represents lantibiotics that are modified by LanM, a bifunctional single enzyme with both dehydratase and cyclase activity. Class III lantibiotics are modified by the single enzyme, LanKC, whereas class IV lantibiotics members are modified by LanL. Nisin, a classic example of LAB lantibiotics, has been used as a food preservative (Delves-Broughton, 1990), but resistance of foodborne pathogens to the bacteriocin has been reported (Ming and Daeschel, 1993; Mazzotta et al., 1997). Advantages such as structural stability, low cytotoxicity, and broad spectrum of activity sustained the search for new lantibiotics (Begley et al., 2009; Montalbán-López et al., 2012; Sandiford, 2015).

Besides the classic culture-based and chromatographic techniques, genome mining has been used recently to predict bacteriocin-related sequences (Nes and Johnsborg, 2004; Begley et al., 2009; Singh et al., 2015). The combination of microbiological, proteomic, and genomic analyses is a promising approach to facilitate and speed up the discovery of novel bacteriocins. Genome-guided mass spectrometry (MS) can be defined as mass spectrometric analysis conducted based on the information obtained from genomic analysis with the goal of expediting the discovery of new antimicrobial peptides (van Heel et al., 2017). Implementing this approach, the current investigation led to the discovery of a new bacteriocinogenic strain, *Lactobacillus paraplantarum* OSY-TC318, and its novel lantibiotic, paraplantaricin TC318. The genome of the bacterium was demarked among closely related strains using comparative genome analysis. A genome-guided mass spectrometric analysis was utilized to rapidly reveal the structure of paraplantaricin TC318. To the best of our knowledge, paraplantaricin TC318 is a novel member of class I lantibiotics, and the bacteriocin is potentially useful in food or medical applications.

## Materials and Methods

### Screening Foods for Antimicrobial-Producing Bacteria

Thirty-three food products were purchased from grocery stores in the United States and Turkey. These included dairy products (kefir, cheddar, blue, Roquefort, and fermented Turkish cheeses), herbs (thyme and mint), spices (cumin, black pepper, red pepper, and spices mix), meat products (Italian salami, fermented sausage, ground meat, and a Turkish sausage known as sucuk), fermented soybean, kimchi, and Turkish beverages (Boza, Salgam, and pickle juice). Food samples (10 g each) were homogenized in 90 ml of 0.1% sterile peptone water using a homogenizer. Tenfold serial dilutions were made from the homogenate, using the same diluent, and a 100-μl aliquot from each dilution was spread onto plate count agar (PCA), de Man, Rogosa and Sharpe (MRS) agar, and potato dextrose agar (PDA) (Oxoid, Thermo Fisher Scientific, Waltham, MA, United States). For aerobic mesophilic bacteria, the inoculated PCA plates were incubated aerobically at 30°C for 72 h. For LAB isolation, the inoculated MRS plates were incubated anaerobically at 30°C for 48 h. For the isolation of yeasts and molds, the inoculated PDA plates were incubated aerobically at 25°C for 5 days. Morphologically different colonies, on incubated plates, were isolated by streaking on the corresponding agar media. Several hundreds of isolates were screened for their ability to produce antimicrobial agents using the soft-agar overlay technique (Guo et al., 2012), with modifications. Briefly, the isolates were inoculated onto MRS agar or tryptic soy agar (TSA; Becton Dickinson, Sparks, MD, United States) and the plates were incubated at 30°C for 24 h. The incubated plates were then overlaid with soft agar media that had been pre-inoculated with *Pediococcus pentosaceus*, *Listeria innocua* ATCC 33090, or *Escherichia coli* K-12, as the indicator bacteria. The soft agar medium was Luria–Bertani broth (Difco, Franklin Lakes, NJ, United States) or MRS broth, both supplemented with 0.75% agar (Thermo Fisher Scientific, St. Louis, MO, United States) and 0.06% CaCO_3_ for neutralizing the acid produced by LAB. After incubation at 30°C for 24 h, the overlaid plates were inspected for inhibition of the indicator strains. Colonies responsible for the inhibition of the indicators were isolated and analyzed further.

### Bacterial Strains and Media

The producer strain, OSY-TC318, was streaked on MRS agar, and its overnight subculture in MRS broth was mixed in 80% sterile glycerol in a 1:1 ratio and stocked at −80°C in the food microbiology laboratory culture collection, The Ohio State University (OSU; Columbus, OH, United States). Selected bacterial strains (Table 1) were cultivated in MRS broth or TSB to test the spectrum of the antimicrobial agent produced by OSY-TC318.

**TABLE 1:** Bacteria tested sensitive^*a*^ to *Lactobacillus paraplantarum* OSY-TC318 crude extract.

Tested bacteria	Diameter of inhibitory area (mm)^*b*^
**Gram-positive (non-spore former)**	
*Enterococcus faecalis* OSU 48	17
*Listeria monocytogenes* California	16
*L. monocytogenes* Ohio (nisin resistant)	15
*L. monocytogenes* OSY-8517	16
*L. monocytogenes* Scott A	16
*L. monocytogenes* V7	16.5
*Listeria innocua*	13
*Micrococcus luteus*	25
*Staphylococcus aureus* ATCC 25923	15
*S. aureus* ATCC 29213	11
*S. aureus* ATCC 6538	14
*S. aureus* OSU 150	13
*Staphylococcus epidermidis* 838	11
*Streptococcus agalactiae*	21
**Gram-positive (spore former)**	
*Bacillus cereus* ATCC 11778	15
*B. cereus* ATCC 12826	16
*B. cereus* ATCC 14579	14
*B. cereus* ATCC 49064	16
*B. cereus* ATCC 33019	12
*Bacillus subtilis* (tetracycline-resistant)	20
**Gram-negative**	
*Enterobacter aerogenes*	12
*Enterobacter liquefaciens*	14
*Escherichia coli* K12	14
*Proteus vulgaris*	15
*Salmonella enterica* ser. Typhimurium	10
*Yersinia enterocolitica*	16.5

*^*a*^Sensitivity was measured as the visible inhibition of growth during the bioassay, as described in the “Materials and Methods” section. ^*b*^Diameter represents the sum of zone of inhibition and well width; the latter is 7 mm.*

### Determination of the Antimicrobial Spectrum of OSY-TC318 Cellular Crude Extract

Preliminary data showed that the OSY-TC318 strain produces an antimicrobial agent when grown on MRS agar plates, but not in MRS broth. Therefore, the OSY-TC318 strain overnight culture in MRS broth was spread-plated onto 300 MRS agar plates, 100 μl culture per plate, followed by incubation at 30°C for 72 h. After incubation, OSY-TC318 cells were harvested using a microscopic slide and the harvested cells mixed with 70% isopropanol in a 1:4 (*w*/*v*) ratio for extracting the antimicrobial agent. After shaking at 200 rpm and 25°C for 3 h, the mixture was centrifuged at 7,710 × *g* for 15 min to remove cell debris. The isopropanol in the extract was evaporated using a vacuum concentrator (Speed-Vac; Savant SPD131DDA, Thermo Fisher Scientific). A bioassay technique was used to determine the antimicrobial activity in the concentrated suspension (crude extract) using the agar well diffusion method (Balouiri et al., 2016) against selected strains (Table 1) as indicators. Briefly, aliquots (10 μl) of the overnight indicator bacterium were transferred into 10 ml of sterile molten soft agar (0.75% agar) of the MRS broth or TSB. The mixture was poured onto a basal TSA agar plate. After the soft agar solidified, wells were made using the wide end of a sterile pipette and 80-μl aliquots of the crude extracts were dispensed in the agar wells. After incubation at 30°C or 37°C overnight, the plates were inspected for the presence of clear inhibitory zones around the wells.

### Testing OSY-TC318 Cellular Crude Extract Against *Clostridium sporogenes* Spores

*Clostridium sporogenes* ATCC 7955 spore suspension was prepared as follows. Stock culture of the bacterium was inoculated in freshly prepared reinforced clostridial medium (RCM) broth and incubated anaerobically at 37°C for 3 days. The resulting culture was streaked on RCM agar and incubated as previously described. Colonies from the RCM agar were transferred to 100 ml of Duncan–Strong sporulation medium, modified for *Clostridium perfringens* (Rhodehamel and Harmon, 2001), followed by anaerobic incubation at 30°C for 7 days. After incubation, the spore-containing medium was centrifuged at 5,857 × *g* for 10 min. The spore pellet was resuspended in 25 ml sterile water followed by a heat shock at 80°C for 25 min. The suspension was re-centrifuged at 5,857 × *g* for 10 min followed by resuspension in 25 ml phosphate-buffered saline (137 mmol/l NaCl, 2.7 mmol/l KCl, 10 mmol/l Na_2_HPO_4_/KH_2_PO_4_, pH 7.4) containing 0.5 mg/ml lysozyme (Sigma-Aldrich, St. Louis, MO, United States). The suspension was incubated at 37°C for 2 h and centrifuged again at 2,510 × *g* for 20 min. The pellet was washed with sterile water and centrifuged at 2,510 × *g* for 20 min, followed by resuspension in sterile water. This final *C. sporogenes* spore suspension contained 7.6 × 10^6^ spore-forming units (SFU)/ml, as counted on RCM agar. The antimicrobial activity of the OSY-TC318 crude extract against *C. sporogenes* spores was determined in sterile polymerase chain reaction (PCR) tubes. Briefly, each tube contained 178 μl freshly prepared RCM broth, 20 μl *C. sporogenes* spore suspension (final concentration at 10^4^ SFU/ml), and 2 μl crude extract. The antimicrobial activity was indicated by observing no turbidity after anaerobic incubation at 37°C for 18 h.

### Identification of the OSY-TC318 Strain Using 16S rDNA Sequencing

The 16S ribosomal RNA (rRNA) gene (16S rDNA) was sequenced to identify the producer isolate, OSY-TC318, as previously described (Weisburg et al., 1991; Yang et al., 2016), with modifications. A single colony of strain OSY-TC318 was cultured in MRS broth at 30°C for 18 h. Aliquot (1 ml) of the broth culture was centrifuged at 16,000 × *g* for 2 min. The supernatant was discarded and 180 μl lysis buffer containing 20 mg lysozyme (Sigma-Aldrich) per milliliter nuclease-free water (Qiagen, Hilden, Germany) was used to resuspend the cell pellet, followed by incubation at 37°C for 2 h. The genomic DNA in the lysed OSY-TC318 cells was extracted and purified using a commercial kit (DNeasy blood and tissue kit; Qiagen, Valencia, CA, United States), following the manufacturer’s protocol. Universal primers, specific for amplifying bacterial 16S rDNA (forward primer: 5′-CCGAATTCGTCGACAACAGAGTTTGATCCTGGCTCAG-3′; reverse primer: 5′-CCCGGGATCCAAGCTTAAGGAGGTG ATCCAGCC-3′) were used in the PCR. The sequence within the 16S rDNA was amplified using DNA polymerase (MyTaq, Bioline, Taunton, MA, United States) under the following thermocycler conditions: (i) an initial incubation at 95°C for 3 min; (ii) 35 cycles of denaturation at 95°C for 15 s, annealing at 52°C for 1 min, and extension at 72°C for 30 s; and (iii) a final extension step at 72°C for 10 min. The PCR product was purified using a commercial DNA purification kit (QIAquick gel extraction kit; Qiagen) following the manufacturer’s instructions. The purified PCR product was sequenced at the OSU Nucleic Acid Shared Resource facility (Columbus, OH, United States). The resulting 16S rDNA sequence was compared to known sequences in the National Center for Biotechnology Information (NCBI) database using Basic Local Alignment Search Tool-Nucleic Acids (BLAST-N; Boratyn et al., 2013).

### Identification of the OSY-TC318 Strain Using MALDI-TOF MS

Identity of the antimicrobial producer, OSY-TC318, was determined at the OSU Medical Center (Columbus, OH, United States) using a matrix-assisted laser desorption/ionization–time of flight mass spectrometry (MALDI-TOF MS) biotyper (Bruker Biotyper; Bruker Daltonics, Billerica, MA, United States) as previously described (Blosser et al., 2016), with modifications. Before the analysis, OSY-TC318 was streaked onto MRS agar and incubated at 30°C for 48 h to obtain well-isolated colonies. The biotyper was calibrated using a bacterial test standard, *E. coli* DH5 alpha (BTS, Bruker Daltonics). A working matrix solution was made of 250 μl standard solvent (50% acetonitrile, 47.5% water, and 2.5% trifluoroacetic acid) and 2.5 mg α-cyano-4-hydroxycinnamic acid crystals (HCCA, Bruker Daltonics). This mixture was vortexed until the crystals were dissolved. Using a wooden toothpick, a single colony was spotted on a clean MBT Biotarget 96-well plate (Bruker Daltonics) and allowed to dry. One microliter of the working matrix solution was added onto the spotted well and allowed to dry. The 96-well plate was loaded into the mass spectrometer (Bruker MicroFlex LT; Bruker Daltonics). Spectra were collected following the method supplied by the equipment’s manufacturer. For the spectral analysis and isolate identification, the biotyper software (MTB Compass 4.1; Bruker Daltonics) was used to match the spectrum of the isolate against the MALDI-TOF MS Bruker library following Bruker’s recommended parameters. Scores between 1.70 and 1.99 were considered appropriate for genus-level identification and those ≥2.0 were considered candidates for species-level identification. Three independent experiments were done to confirm the identity of the antimicrobial-producing bacterium, OSY-TC318.

### Genome-Based Taxonomy of the OSY-TC318 Strain

The genome of OSY-TC318 was sequenced previously and the draft genome sequence was deposited in the GenBank database under accession number SEHH00000000.1 (Hussein et al., 2019). For the purpose of accurately classifying the OSY-TC318 strain and exploring its phylogenetic relationship with closely related strains, the following genomic analysis was executed. A genomic-based comparison was conducted between the OSY-TC318 draft genome and 11 publicly accessible genomes, announced as *L. paraplantarum*, in the NCBI genome database (Table 2). The Genome Taxonomy Database Toolkit (GTDB-Tk v1.0.2) was used for assigning a taxonomic classification for the 12 investigated genomes, including OSY-TC318, based on: a) placement in a domain-specific reference tree representing 23,471 bacterial genomes and b) calculating their average nucleotide identity (ANI) to a reference genome considering 95% ANI as a species circumscription radius (Chaumeil et al., 2019; Parks et al., 2019). In the 12 query genomes, GTDB-Tk identified genes using a prokaryotic dynamic programming gene-finding algorithm, PRODIGAL v2.6.2 (Hyatt et al., 2010). Out of these genes, 120 bacterial marker genes, encoding ubiquitous single-copy proteins, were identified and aligned using the profile hidden Markov models (profile HMMs) search tool, HMMER v3.1 (Eddy, 2011; Parks et al., 2018), for phylogenetic inference. From the concatenated multiple sequence alignment of the marker genes, the phylogenetic placement tool pplacer v1.1 (Matsen et al., 2010) was used for maximum-likelihood placement of the query genomes into the GTDB-Tk reference tree. Finally, GTDB validated the species assignment by calculating the ANI of the query genomes, based on *L. paraplantarum* DSM 10667 (GCA_001435655.1) as a GTDB species representative, using the ANI determination tool FastANI v2.0 (Jain et al., 2018). For data visualization, a phylogenetic sub-tree which contains the query genomes was isolated from the GTDB-Tk reference tree and viewed using the phylogenetic trees visualization and manipulation tool (Interactive Tree Of Life, iTOL, v4) (Letunic and Bork, 2019). AnnoTree v1.2, a tool used for the visualization of genome annotations across large phylogenetic trees, was used to locate *L. paraplantarum* across 27,000 bacterial genomes phylogenetic trees, derived from the GTDB database (Mendler et al., 2019). Additionally, the query genomes were compared visually against the OSY-TC318 genome based on the genomic sequence alignment using the BLAST ring image generator, BRIG (Alikhan et al., 2011).

**TABLE 2:** Genomic features and average nucleotide identity (ANI) of *Lactobacillus paraplantarum* genomes, including OSY-TC318 (this study), when compared against *L. paraplantarum* DSM 10667 (GCF_001435655.1) as a reference genome.

NCBI^*a*^ strain identifiers	Source	Assembly accession	Genome size (Mb)	GC%	Genes	Proteins	ANI%
DSM 10667	Beer contaminant, France	GCA_001435655.1	3.39575	43.7	3,280	3,094	100
NBRC 107151	Beer contaminant, France	GCA_007991915.1	3.33087	43.7	3,246	3,072	99.99
DSM 10667	Beer contaminant, France	GCA_003641145.1	3.36853	43.9	3,328	3,098	99.94
AS-7	Fruits and vegetables, Pakistan	GCA_003045685.1	3.00744	44.1	2,890	2,730	99.94
AY01	Ewes milk, China	GCA_000469115.1	3.31597	43.7	3,202	3,021	99.73
D2-1	Unknown	GCA_002897175.1	3.52146	43.6	3,531	3,275	99.57
Lp109	Cured beef, China	GCA_003609655.1	3.38074	43.7	3,372	3,120	99.54
OSY-TC318	Cheese, Turkey	GCA_004330485.1	3.58749	43.4	3,474	2,846	98.23
L-ZS9	Fermented meat, China	GCA_001443645.1	3.13973	44.0	2,990	2,792	97.66
L-ZS9	Fermented meat, China	GCA_000758145.1	3.11972	43.9	2,947	2,807	97.64
KMB-599	Sheep milk, Slovakia	GCA_003346405.1	3.17349	43.9	3,058	2,865	97.59
CRL 1905	Quinoa sourdough, Argentina	GCA_001660655.1	1.80977	44.7	1,681	1,622	Not applicable^a^

*^*a*^NCBI, National Center for Biotechnology Information. ^*b*^See text for detailed explanation.*

### Mining the Genomes of OSY-TC318 and Closely Related Strains for Bacteriocin Sequences

Considering the relatedness between OSY-TC318 and the 11 *L. paraplantarum* strains, genomes of the 12 strains were mined for genes related to bacteriocin biosynthesis using the antibiotics and secondary metabolite analysis shell software antiSMASH v4.1.0 (Blin et al., 2018) and the web-based bacteriocin genome mining tool BAGEL v4.0 (Walsh et al., 2011). After identifying a putative novel lantibiotic gene cluster uniquely in the OSY-TC318 draft genome, NCBI-BLASTP was used to align the cluster genes with related sequences in the NCBI database (Boratyn et al., 2013) for their function prediction. Knowing the potential functions of the cluster genes has led to the prediction of the putative lantibiotic, designated paraplantaricin TC318, amino acid sequence, and its biosynthetic pathway. The theoretical molecular mass of the lantibiotic was calculated from its predicted amino acid sequence using the proteomics analysis tool Protein Prospector v5.22.0 (Knudsen and Chalkley, 2011).

### Liquid Chromatographic Purification of Paraplantaricin TC318 and Analysis Using MALDI-TOF MS

A high-performance liquid chromatography (HPLC) system (Hewlett Packard 1050, Agilent Technologies, Palo Alto, CA, United States) was used to purify the antimicrobial agent produced by the OSY-TC318 strain from its cellular crude extract. The stationary phase was a reversed-phase C_18_ column (Discovery BIO C_18_ column, 25 cm × 4.6 mm I.D., 5 μm, Supelco, Bellefonte, PA, United States). The mobile phase consisted of (A) acetonitrile with 0.1% trifluoroacetic acid (TFA) and (B) HPLC-grade water with 0.1% TFA. For each run, aliquots (100) of the crude extract were loaded and separated on the column by a linear gradient of solvent A from 0 to 100% over 30 min followed by 100% solvent A for 5 min at a flow rate of 1.0 ml/min. Elution was monitored using a UV detector at a wavelength of 220 nm. Fractions were collected at a rate of one fraction per minute (Fraction Collector II; Waters Cooperation, Milford, MA, United States). Fractions with the same retention time from multiple runs were combined and dried using a vacuum concentrator (Speed-Vac). The resulting dry fractions were dissolved in 70% acetonitrile and the antimicrobial activity of each fraction was tested against *P. pentosaceus* as an indicator. When spotted on the surface of the inoculated soft agar plate, the solvent (70% acetonitrile), which dissipated quickly, did not show antimicrobial activity against the indicator bacterium after overnight incubation. The multiple HPLC run fractions, containing the semi-purified antimicrobial agent, were further purified by re-injection into the HPLC using the same conditions described previously. To confirm that the antimicrobial activity is caused by the putative lantibiotic, paraplantaricin TC318, active fractions were initially analyzed using a MALDI-TOF/TOF mass spectrometer (UltrafleXtreme; Bruker Daltonics), operated in reflection positive ion mode, at the Campus Chemical Instrument Center (CCIC), OSU (Columbus, Ohio, United States). The matrix was HCCA (Bruker Daltonics) which was dissolved in acetonitrile with 0.1% TFA in water (1:1, *v*/*v*) to obtain a saturated matrix solution. The HPLC fractions with antimicrobial activity were mixed with the matrix in a ratio of 1:5 (*v*/*v*). One microliter of the previous mixture was spotted on the MALDI stainless steel plate. The spectral masses, in the range of *m*/*z* 500–5,000, were detected. A neodymium-doped yttrium aluminum garnet laser (Yag/Nd laser), at 351 nm, was set at 5% higher than the threshold level to minimize fragmentation. The mass spectrometer was calibrated using a peptide calibration standard (Standard II; Bruker Daltonics). The spectra were analyzed using commercial analysis software (Flex; Bruker Daltonics). The HPLC fractions that showed a peak with an average mass congruent to the calculated mass of paraplantaricin TC318 were selected for accurate mass determination during subsequent analysis.

### FT-ICR-MS Analysis

Accurate mass determination and sequencing of paraplantaricin TC318 required analysis by Fourier transform ion cyclotron resonance mass spectrometry (FT-ICR-MS). Active, concentrated, and pure HPLC fractions containing paraplantaricin TC318 were subjected to FT-ICR-MS analysis (Bruker 15 Tesla solariXR FT-ICR mass spectrometer; Bruker Daltonics) at CCIC, OSU (Columbus, OH, United States). The spectrometer, coupled with a MALDI or electron spray ionization (ESI) source, was used with a 4M transient that provided the required resolution for accurate mass measurement (<1 ppm error). The MALDI source was used for the purpose of determining the accurate mass of the singly charged ions and for peptide sequencing, whereas the ESI source was used for determining the accurate mass of the doubly charged ions. For MALDI analysis, the samples were mixed with the HCCA MALDI matrix solution in a ratio of 1:5 (*v*/*v*) and 1 μl of the mixture was spotted on the MALDI stainless steel plate. The mass spectrometer was calibrated using a peptide calibration standard (Bruker Daltonics). A neodymium-doped yttrium aluminum garnet laser (Yag/Nd, 351 nm) was used for ionization with varying laser powers of 20–25% to ionize/desorb the peptide ions. Ions were detected in the range of *m*/*z* 500–4,000. Additional quadrupole collision-induced dissociation (QCID) MS/MS analysis was performed at 80-eV collision energy (with a collision gas of argon) and 40% laser power to fragment the singly charged peptide and obtain its amino acid sequence. For the ESI source, the original HPLC fractions were diluted 10-fold in a solvent made of water and acetonitrile, at a 1:1 ratio, and containing 0.1% TFA. The diluted solution was sprayed using standard ESI conditions (ESI spray voltage = 4,500 V, capillary voltage = 250 V, capillary temperature = 180°C, and flow rate = 2.5 μl/min).

## Results

### Isolation of Antimicrobial-Producing Strain and the Inhibition Spectrum of Its Cellular Crude Extract

Several hundred isolates, obtained from 33 food products, were screened for their antimicrobial activity; among these, a few isolates showed antimicrobial activity. A Turkish cheese isolate, designated OSY-TC318, showed strong inhibition against the indicator bacterium, *P. pentosaceus*. Subsequently, OSY-TC318 was subjected to further analyses to determine its identity and characterize potential antimicrobial agents. The OSY-TC318 cell-free crude extract showed a wide range of antimicrobial activity when tested against selected bacterial pathogenic and spoilage strains such as *Bacillus cereus*, *Enterococcus faecalis*, *Listeria monocytogenes*, *Salmonella* Typhimurium, and *Staphylococcus aureus* (Table 1). Importantly, the OSY-TC318 cell-free crude extract inhibited the germination or outgrowth of *C. sporogenes* spores; therefore, the strain OSY-TC318 was selected for comprehensive genomic and proteomic analyses.

### Comparing 16S rDNA Sequencing and MALDI-TOF MS Biotyping as Methods for Rapid Identification of OSY-TC318 Isolate

Due to the promising antimicrobial potential of OSY-TC318, its identity was investigated using multiple approaches. Sequencing the 16S rRNA gene of the OSY-TC318 strain resulted in 95% identity with *Lactobacillus plantarum* strains SM2, gp106, and gp108 and also 95% identity with *L. paraplantarum* strains JCM12533, MRS8, and FH3. Based on these results, it was concluded that the 16S rDNA sequencing approach was insufficient for the accurate identification of the OSY-TC318 strain. Using MALDI-TOF MS-based biotyping, it was found that the OSY-TC318 spectrum had a first-best match with *L. paraplantarum* with a score of 2.24; the second-best match was *L. plantarum* with score of 2.17. Therefore, OSY-TC318 was preliminary identified as *L. paraplantarum*. However, genomic analysis was required for the accurate taxonomic classification of the OSY-TC318 strain and for distinguishing it among closely related strains of *L. paraplantarum*.

### Genome-Based Taxonomy of OSY-TC318 and Its Demarcation Among Closely Related Strains Using Comparative Genomic Analysis

**FIGURE 1:**
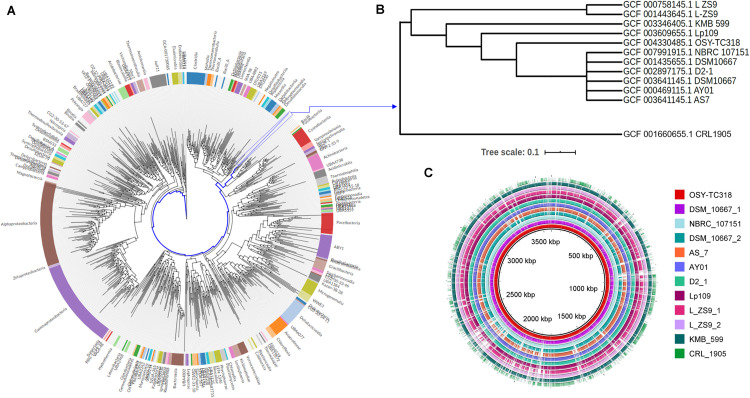
Phylogenetic relationship and genomic-based comparison between *Lactobacillus paraplantarum* OSY-TC318 and closely related genomes. **(A)** Placement of *L. paraplantarum* OSY-TC318 in a phylogenetic tree that represents 27,000 bacterial genomes. **(B)** Placement of *L. paraplantarum* OSY-TC318 among closely related genomes of the species. **(C)** BLAST Ring Image Generator (BRIG) diagram comparing 11 query genomes, announced as *L. paraplantarum* on NCBI, with OSY-TC318 considered as a reference genome. In the BRIG diagram **(C)**, *L. paraplantarum* genomes are arranged from *inner* to *outer circle* as follows: OSY-TC318, DSM_10667_1, NBRC_107151, DSM_10667_2, AS_7, AY01, D2_1, Lp109, L_ZS9_1, L_ZS9_2, KMB_599, and CRL_1905. Genomic regions with 0% identity with the reference genome are indicated as *blank spaces*.

Using comparative genomic approach, the OSY-TC318 strain along with 10 other known *L. paraplantarum* genomes were classified, based on placement in the GDTB reference tree (Figures 1A,B) and ANI% (Table 2), as *L. paraplantarum*. In addition to its phylogeny, GTDB-Tk revealed that *L. paraplantarum* OSY-TC318 has an ANI of 98.23% with *L. paraplantarum* DSM 10667 (GCF_001435655.1). Although the CRL 1905 genome is announced on NCBI as *L. paraplantarum*, GTDB taxonomy assigned CRL 1905 to the genus *Lactobacillus* based on the topological placement and showed that it has an ANI of 98.65% with *Lactobacillus rhamanosus* DSM 20021(GCA_000615245.1). When aligning OSY-TC318 with the queried genomes, the blank spaces represent the genomic regions with 0% identity (Figure 1C); this shows how different this newly discovered strain is from other *L. paraplantarum* strains. The phylogeny, the ANI, and the genomic sequence alignment analyses confirmed the identity of *L. paraplantarum* OSY-TC318 and also distinguished it from the previously announced *L. paraplantarum* genomes. One of the most important distinguishing features was the presence of a unique lantibiotic gene cluster in *L. paraplantarum* OSY-TC318, as will be described later.

### Genome Mining for Bacteriocin Sequences and the Analysis of a Novel Lantibiotic Biosynthetic Gene Cluster

**FIGURE 2:**
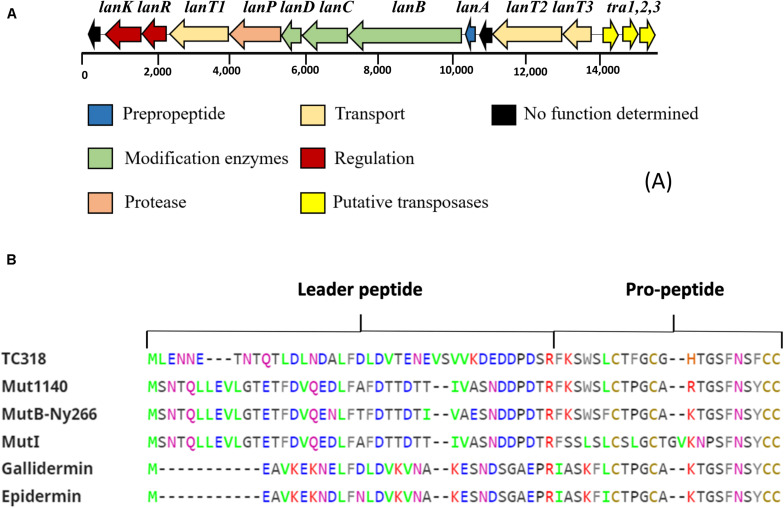
Bioinformatics analysis of the novel lantibiotic paraplantaricin TC318. **(A)** Genetic organization of paraplantaricin TC318 biosynthetic gene cluster. **(B)** Primary sequence alignment of lantibiotic TC318 amino acid residues with the sequences of mutacin 1140, mutacin B-Ny266, mutacin I gallidermin, and epidermin using ClustalW tool (http://www.ebi.ac.uk/tools/clustalw2). Mutacin 1140 is the most related lantibiotic to TC318.

A comparative genome mining for bacteriocin sequences in *L. paraplantarum* OSY-TC318 and the other 11 closely related genomes revealed several plantaricin genetic clusters in all the investigated genomes. Common plantaricin genetic clusters *(pln)* detected were *plnABCD*, *plnJKLR*, and *plnEFI*. Importantly and uniquely, a type I lantibiotic biosynthetic gene cluster was detected in the *L. paraplantarum* OSY-TC318 genome. The identified biosynthetic cluster of the putative lantibiotic, paraplantaricin TC318, comprised 11 open reading frames (ORFs). The proposed function of each ORF, length, closest homolog, percent identity, and the percent query coverage are shown in Table 3. The paraplantaricin TC318 biosynthetic gene cluster includes the prepropeptide structural gene (*lanA*), modification enzyme genes (*lanB*, *lanC*, and *lanD*), a protease enzyme gene (*lanP*), three secretion ABC-type transporter and self-immunity genes (*lanT1*, *lanT2*, and *lanT3*), two regulatory genes (*lanK* and *lanR*), and a gene with an unknown function (Figure 2A).

**TABLE 3:** Open reading frame (ORF) analysis of the putative paraplantaricin TC318 biosynthetic gene cluster and flanking genes.

ORF no.	Proposed function	Length (amino acids)	GenBank accession number; closest homolog (NCBI database accessed on 7/11/2018)	Percent identity (percent query cover)
1	No function determined	101	WP_063487862.1, hypothetical protein [*Lactobacillus plantarum*]	93 (91)
2	LanK (sensor histidine kinase)	327	WP_062688923.1, sensor histidine kinase [*Lactobacillus plantarum*]	99 (100)
3	LanR (transcriptional regulatory protein)	225	WP_062688921.1, DNA-binding response regulator [*Lactobacillus plantarum*]	100 (100)
4	LanT1 (ABC transporter ATP-binding protein)	547	WP_062688919.1, ABC transporter ATP-binding protein [*Lactobacillus plantarum*]	100 (100)
5	LanP (leader peptide processing protease)	461	WP_032531395.1, peptidase S8 lantibiotic specific protease[*Streptococcus mutans*]	44 (97)
6	LanD (decarboxylation enzyme)	186	WP_002304546.1, Lantibiotic biosynthesis protein [*Streptococcus mutans*]	49 (94)
7	LanC (lanthionine synthetase C family protein)	437	WP_002289980.1, lantibiotic epidermin biosynthesis protein EpiC [*Streptococcus mutans*]	99 (100)
8	LanB (lantibiotic dehydratase domain protein)	1,005	AAG48566.1, *MutB [Streptococcus mutans*]	32 (98)
9	LanA (prepropeptide of TC318)	62	WP_002268802.1, gallidermin/nisin family lantibiotic [*Streptococcus mutans*]	57 (87)
10	No function determined	114	WP_062688908.1, hypothetical protein [*Lactobacillus plantarum*]	100 (100)
11	LanT2 (ABC transporter, ATP-binding protein)	649	WP_062688906.1, ABC transporter permease [*Lactobacillus plantarum*]	99 (100)
12	LanT3 (ABC transporter, ATP-binding protein)	246	WP_062688904.1, ABC transporter ATP-binding protein [*Lactobacillus plantarum*]	100 (100)
13	Tra1 (transposase)	95	ERO39513.1, putative transposase [*Lactobacillus plantarum* WJL]	74 (100)
14	Tra2 (transposase)	76	WP_076644604.1, transposase [*Lactobacillus plantarum*]	93 (100)
15	Tra3 (transposase)	139	WP_016527020.1, transposase [*Lactobacillus plantarum*]	90 (97)

**FIGURE 3:**
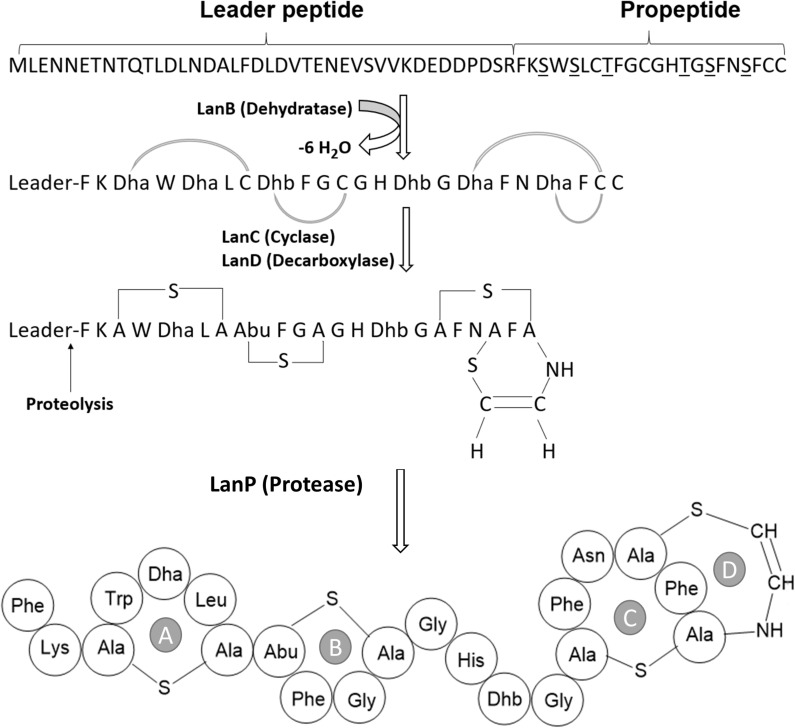
Proposed posttranslational modifications and structure of paraplantaricin TC318. TC318 contains four thioether rings (A–D). *Dha*, dehydroalanine; *Dhb*, dehdrobutyrine.

The proposed paraplantaricin TC318 biosynthetic mechanism (Figure 3) starts with the expression of the structural gene to make the prepropeptide. The prepropeptide consists of a propeptide at the C-terminus and a leader peptide at the N-terminus. Some of the proposed functions of the leader peptide are signaling for peptide export and serving as a scaffold for assisting posttranslational modifications, including the cleavage of the prepropeptide by a peptidase (Chatterjee et al., 2005; Jack et al., 2008). The specific posttranslational modifications of paraplantaricin TC318 are introduced by enzymes encoded in the biosynthetic gene cluster. Based on the sequencing of this biosynthetic gene cluster, it can be inferred that these enzymes catalyze the dehydration (Lan B) and cyclization (Lan C) of the serine and threonine residues in the propeptide that can condense with the neighboring cysteine residue, leading to the formation of lanthionine and methyllanthionine (thioether) bridges, respectively. The enzyme encoded by *lanD* may catalyze the decarboxylation of the C-terminal cysteine residue, forming the C-terminal S-[(*Z*)-2-aminovinyl]-D-cysteine (AviCys) residue (Chatterjee et al., 2005). After the dehydration and the cyclization, the leader peptide is removed by the protease as the last step in the biosynthesis (Sahl and Bierbaum, 1998). Based on the genomic analysis, the predicted amino acid sequence of paraplantaricin TC318 consisted of 22 residues that differ from the mutacin 1140 sequence by four amino acids (Figures 2B, 3). The predicted chemical formula of paraplantaricin TC318 is C_106_H_133_N_27_O_22_S_4_, which corresponds to a monoisotopic neutral molecular mass of 2,263.900 Da.

### Purification of Paraplantaricin TC318 and Structural Elucidation Using FT-ICR-MS

**FIGURE 4:**
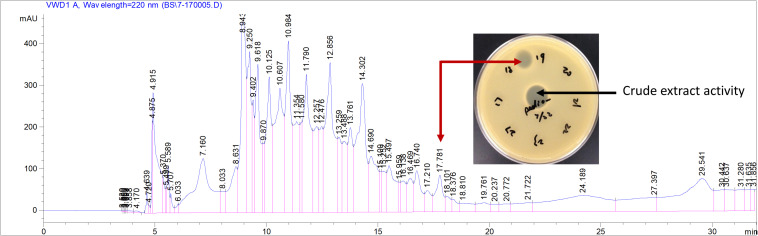
Purification of paraplantaricin TC318 from crude extract using liquid chromatography. *Red arrow* indicates the single peak showing antimicrobial activity against an indicator strain, *Pediococcus pentosaceus*.

**FIGURE 5:**
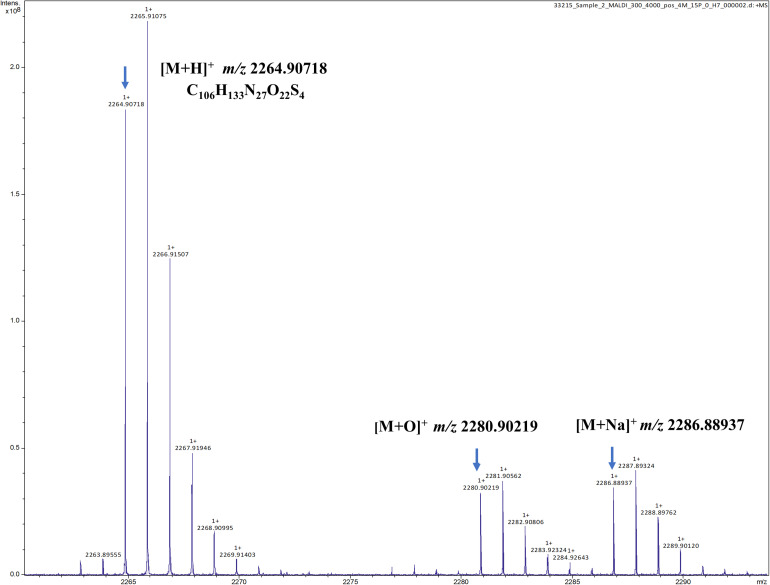
Fourier transform ion cyclotron resonance mass spectrometry/matrix-assisted laser desorption/ionization (FT-ICR-MS/MALDI) analysis of the singly charged ions ([M + H]^+^, [M + O]^+^, and [M + Na]^+^) from intact paraplantaricin TC318 with the observed *m*/*z* of 2,264.90718, 2,280.90219, and 2,286.88937, respectively.

**FIGURE 6:**
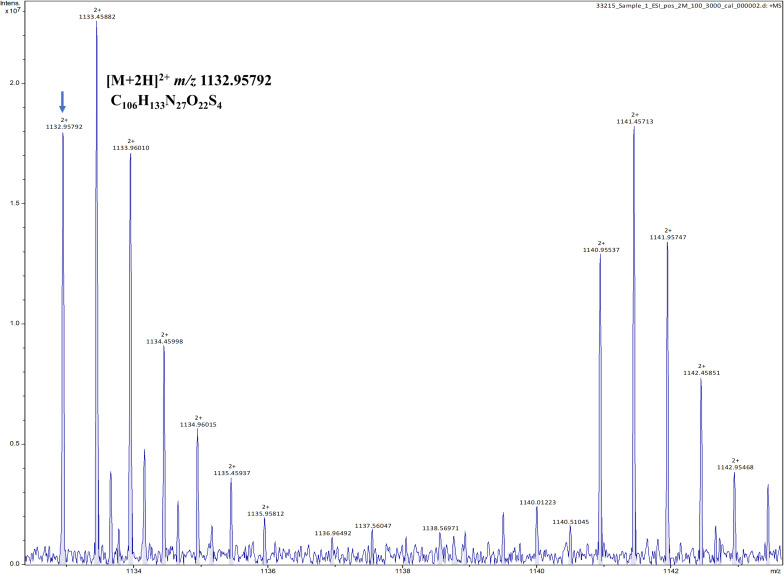
Fourier transform ion cyclotron resonance mass spectrometry/electron spray ionization (FT-ICR-MS/ESI) analysis of paraplantaricin TC318 doubly charged ion ([M + 2H]^2+^) with an observed *m*/*z* of 1,132.95792.

To verify the predicted structure of the lantibiotic produced by *L. paraplantarum* OSY-TC318, the compound was separated and purified using reversed-phase HPLC. During a 30-min HPLC run of 0–100% acetonitrile gradient, the active antimicrobial agent was eluted at a retention time of 17.8 min, which was detected using the antimicrobial activity assay of eluted fractions. A representative HPLC chromatograph of the crude extract and the corresponding active fraction is presented in Figure 4. For accurate mass determination, the purified HPLC fraction was analyzed using FT-ICR-MS, coupled with a MALDI or ESI source. When a MALDI source was used, the results showed peaks of the singly charged ions ([M + H]^+^, [M + O]^+^, and [M + Na]^+^) of the intact paraplantaricin TC318, with *m*/*z* values of 2,264.90718, 2,280.90219, and 2,286.88937, respectively (Figure 5). Therefore, the accurate monoisotopic mass of the paraplantaricin TC318 singly charged ion, [M + H]^+^, is *m*/z 2,264.90718, a value that matched the previously calculated theoretical molecular mass of 2,263.900, after subtracting the hydrogen ion, with 0.1 ppm error. Using the ESI source, the FT-ICR-MS results showed a doubly charged paraplantaricin TC318 ([M + 2H]^2+^) with an accurate *m*/*z* of 1,132.95792; this matched the corresponding theoretical calculated *m*/*z* of 1,132.957343 with 0.5 ppm error, as shown in Figure 6.

For paraplantaricin TC318 sequencing, the precursor peak of the peptide singly charged ion, [M + H]^+^, was isolated and fragmented using QCID MS/MS analysis. As shown in Supplementary Table S1 and Supplementary Figure S1, a series of MS/MS product ions were observed which matched the predicted structure based on the genomic analysis, as shown in Figure 3 and Supplementary Figure S2. Therefore, the biosynthetic pathway and the structure of the new paraplantaricin TC318 lantibiotic were confirmed based on the genomic analysis combined with MS analysis. However, the thioether ring structure made it difficult for complete fragmentation by QCID.

## Discussion

The discovery of a new beneficial antimicrobial agent is a tedious and time-consuming task, a fact consistent with the experience of the investigators of the current study (He et al., 2007; Guo et al., 2012; Yang et al., 2016). The urgency to speed up the discovery of new antimicrobials prompted these investigators to benefit from the advances in genomic and mass spectrometric analyses to accomplish this goal. The efficient approach implemented in this study led to the discovery of paraplantaricin TC318 from a LAB isolated from cheese. Successful discovery of a new antimicrobial agent not only involves its characterization and structural elucidation but also requires accurate identification of the producer. Considering that antimicrobial production is rare within a given species and is highly strain-dependent, it is critical to identify the producer to the infraspecies level. The following discussion illustrates the approach followed in this study for identifying the antimicrobial-producing isolate to the stain level.

Lactic acid bacteria include many genera, with *Lactobacillus* encompassing the largest group of species that have many applications in food and human health industries (Giraffa et al., 2010; Mokoena, 2017). Despite the commercial value of LAB as dairy starters and probiotics, the use of inaccurate identification methods, such as those based on phenotypic traits, has led to mislabeled strains in scientific publications, publicly available genetic sequences, and commercial products (Huys et al., 2006; Huang et al., 2018). Molecular methods have been developed and used for LAB identification, with 16S rRNA gene sequencing as the most common (Clarridge, 2004). Results of the 16S rRNA gene sequencing for species of the *L. plantarum* group (*L. plantarum*, *L. paraplantarum*, *Lactobacillus fabifermentans*, and *Lactobacillus pentosus*) may show 99% similarity, making it difficult to discriminate among these species using this method (Torriani et al., 2001; Huang et al., 2010). Moreover, using the 16S rRNA gene or any other single phylogenetic marker gene is inadequate to obtain accurate phylogenetic relationships (Parks et al., 2018).

Sequencing the 16S rRNA gene was inadequate to accurately identify OSY-TC318; the results showed that it has the same identity percentage with both *L. plantarum* and *L. paraplantarum*. Biotyping with MALDI-TOF MS is a simple, rapid, and relatively inexpensive method that can be used to identify bacteria based on the mass spectra of highly abundant ribosomal proteins (Ghyselinck et al., 2011). Using this method, the OSY-TC318 isolate was identified as *L. paraplantarum*. Currently, whole-genome sequencing is considered an official source for taxonomic information (Whitman, 2016). In the current study, genome-based taxonomy was used to assign the taxon *L. paraplantarum* to the OSY-TC318 isolate. Comparative genomics, including phylogenetic relationship analysis, ANI calculation, and visualization of the genomic sequence alignments, made it possible to distinguish the genome *L. paraplantarum* OSY-TC318 from 10 closely related genomes and to exclude the *L. paraplantarum* CRL 1905 genome from this group. The constructed phylogeny was based on the concatenation of 120 ubiquitous bacterial single-copy proteins to obtain a higher taxonomic resolution superior to that obtained from the 16S rRNA gene sequence (Parks et al., 2018). These analyses revealed the novelty of *L. paraplantarum* OSY-TC318 and demarked it among closely related strains.

The discovery of new antimicrobial agents, particularly bacteriocin, is a tedious and time-consuming task particularly when a culture-based screening approach is followed. The task is hampered further by the lack of knowledge about the specific conditions or triggers required for the bacteriocin expression, such as nutrients, pH, and temperature (Zhu et al., 2014). Additional complications result from the scarcity of bacteriocin production by the producer strain (Sandiford, 2015) and the necessity of identifying the chemical structure of the putative antimicrobial compound to exclude the unspecific antimicrobial agents and assure the novelty of the investigated bacteriocin (Montalbán-López et al., 2012). Recently, the emergence of genome mining tools has expanded the knowledge about bacteriocins and their biosynthetic mechanisms (Sandiford, 2017); however, verification using culture techniques and proteomic analysis is still needed to validate the genomic analysis output and to confirm the putative bacteriocin expression and biological activity (Collins et al., 2018). Therefore, an efficient combination of culture-based, genomic, and proteomic analyses is required for a time-saving bacteriocin discovery. In the current study, we conducted a genome-guided MS approach that led to elucidating the structure and characterizing the biosynthetic mechanism of paraplantaricin TC318.

In addition to the structural gene (*lanA*), the lantibiotic operon also includes protein-encoding genes that carry out the posttranslational modification (*lanB*, *lanC*, *lanM*, *lanKC*, or *lanL*), the proteolytic processing for cleaving the leader sequence (*lanP*), transportation of the propeptide (*lanT*), and the immunity (*lanI* and *lanFEG*) (Bierbaum and Sahl, 2009; Field et al., 2015; Repka et al., 2017). Mining of the bacteriocin sequences was conducted not only in the OSY-TC318 genome but also in the 11 related *L. paraplantarum* genomes. Various *pln* operons associated with *L. plantarum*, including *plnABCD*, *plnJKLR*, and *plnEFI*, were found in all the query genomes (Diep et al., 1994; Kleerebezem et al., 2003; Maldonado et al., 2004; Rojo-Bezares et al., 2008). Additionally, a unique class I lantibiotic biosynthetic gene cluster was identified in the *L. paraplantarum* OSY-TC318 genome. The cluster includes *lanA*, *lanB*, *lanC*, and *lanD*, three *lanT*, *lanP*, *lanK*, and *lanR* (Figure 2B). Buchman et al. (1988) suggested that the ability of lantibiotic production is dispersed among Gram-positive bacteria by the transfer of lantibiotic-encoding mobile elements such as plasmids or transposons. The presence of transposase genes *tra1*, *tra2*, and *tra3* adjacent to the biosynthetic gene cluster of paraplantaricin TC318 (Figure 2B) suggests that its genetic code may have originated from an ancestral transposon (Chen et al., 1999).

The analysis of the propeptide sequence showed that mutacin 1140, a member of the epidermin group in class I lantibiotic, is the most closely related to paraplantaricin TC318 as both share 18 of their 22 amino acids. The epidermin group is characterized by the presence of one dehdrobutyrine (Dhb), one methyllanthionine, and two lanthionine residues besides the unusual ring structure containing S-[(*Z*)-2-aminovinyl]-D-cysteine (AviCys; Chatterjee et al., 2005). Besides mutacin 1140, gallidermin, mutacin B-Ny266, and mutacin I are other natural variants to epidermin which share the four thioether bridges. Epidermin was isolated from *Staphylococcus epidermidis* Tü 3298 (Allgaier et al., 1985) and gallidermin was isolated from *Staphylococcus gallinarum* Tü 3928 (Kellner et al., 1988). Mutacin 1140, mutacin B-Ny266, and mutacin I were isolated from *Streptococcus mutans* JH1140, Ny266, and CH43, respectively (Mota-Meira et al., 1997; Qi et al., 2000; Smith et al., 2000). To the best of our knowledge, the current study is the first report of a mutacin variant produced by a *Lactobacillus* sp.

*L. paraplantarum* OSY-TC318 produced paraplantaricin TC318 in solid media only. A similar behavior was observed in mutacin 1140, which was explained by the inhibition of structural gene transcription by the mature lantibiotic. Hillman et al. (1998) hypothesized that growing the producer on a solid medium allows the diffusion of the lantibiotic away from the inoculated cells and increases the synthesis time before reaching an inhibitory concentration. To produce the paraplantaricin TC318 crude extract, producer cells were grown and harvested from MRS agar plates followed by extraction using 70% isopropanol. Bacteriocins have antagonistic activity against bacteria of the same species, thus exhibiting a narrow spectrum or, across several genera, exerting a broad spectrum (Cotter et al., 2005). The antimicrobial spectrum of the cell-free crude extract, containing paraplantaricin TC318, showed that it has a wide range of activity against pathogenic and spoilage bacteria (Table 1).

The FT-ICR-MS analysis was executed to elucidate the structure of the purified paraplantaricin TC318, and the results strongly supported the structure predicted by genomic analysis. The paraplantaricin TC318 propeptide consists of 22 amino acids (Phe-Lys-Dha-Trp-Dha-Leu-Cys-Dhb-Phe-Gly-Cys-Gly-His-Dhb-Gly-Dha-Phe-Asn-Dha-Phe-Cys-Cys), as shown in Figure 3. The proposed structure of paraplantaricin TC318 includes four thioether rings, and this makes it resemble members of class I lantibiotics. The thioether ring contains a single sulfur atom and links two amino acids through their β-carbons. The fragmentation was achieved between the rings during the FT-ICR-MS analysis. Despite the lack of complete fragmentation, the chemical formula, the calculated mass, and the observed mass of the fragments confirmed the structure of the rings. In future studies, nuclear magnetic resonance spectroscopic analysis could help in revealing the complete chemical structure of the new lantibiotic compound. Further analyses are planned to determine paraplantaricin TC318’s tertiary structure, mode of action, cytotoxic effect on mammalian cells, and its applicability in various products.

In conclusion, genomic and proteomic analyses were efficiently combined to identify accurately the new *L. paraplantarum* OSY-TC318 strain and to elucidate the structure of its novel lantibiotic, paraplantaricin TC318. The antimicrobial activity of the paraplantaricin TC318 crude extract against a wide range of pathogenic and spoilage bacteria supports its potential usefulness in food and medical applications.

## Data Availability Statement

The raw data supporting the conclusions of this article will be made available by the authors, without undue reservation.

## Author Contributions

AY designed and oversaw the project. IO isolated the producer strain from cheese. XY sequenced the producer genome. EH discovered the lantibiotic gene cluster and developed the biosynthetic pathway. WH tested antimicrobial spectrum, conducted MALDI-TOF biotying, ran comparative genomic analysis, and drafted the manuscript. BL, EH, XY, and WH optimized HPLC purification. ÁS and WH determined the lantibiotic sequence using mass spectrometry techniques. EH and AY contributed to the writing of the manuscript. All authors read and approved the manuscript.

## Conflict of Interest

The authors declare that the research was conducted in the absence of any commercial or financial relationships that could be construed as a potential conflict of interest.

## References

[B1] Abee T. (1995). Pore-forming bacteriocins of Gram-positive bacteria and self-protection mechanisms of producer organisms. *FEMS Microbiol. Lett.* 129 1–9. 10.1016/0378-1097(95)00137-T 7781983

[B2] Alikhan N. F. Petty N. K. Ben Zakour N. L. Beatson S. A. (2011). BLAST ring image generator (BRIG): simple prokaryote genome comparisons. *BMC Genomics* 12:402. 10.1186/1471-2164-12-402 21824423 PMC3163573

[B3] Allgaier H. Jung G. Werner R. G. Schneider U. Zähner H. (1985). Elucidation of the structure of epidermin, a ribosomally synthesized, tetracyclic heterodetic polypeptide antibiotic. *Angew. Chem. Int. Ed. Engl.* 24 1051–1053. 10.1002/anie.198510511

[B4] Balouiri M. Sadiki M. Ibnsouda S. K. (2016). Methods for in vitro evaluating antimicrobial activity: a review. *J. Pharm. Anal.* 6 71–79. 10.1016/j.jpha.2015.11.005 29403965 PMC5762448

[B5] Begley M. Cotter P. D. Hill C. Ross R. P. (2009). Identification of a novel two-peptide lantibiotic, lichenicidin, following rational genome mining for LanM proteins. *Appl. Environ. Microbiol.* 75 5451–5460. 10.1128/AEM.00730-09 19561184 PMC2737927

[B6] Bierbaum G. Sahl H. G. (2009). Lantibiotics: mode of action, biosynthesis and bioengineering. *Curr. Pharm. Biotechnol.* 10 2–18. 10.2174/138920109787048616 19149587

[B7] Blin K. Wolf T. Chevrette M. G. Lu X. Schwalen C. J. Kautsar S. A. (2018). antiSMASH 4.0-improvements in chemistry prediction and gene cluster boundary identification. *Nucleic Acids Res.* 45 W36–W41. 10.1093/nar/gkx319 28460038 PMC5570095

[B8] Blosser S. J. Drake S. K. Andrasko J. L. Henderson C. M. Kamboj K. Antonara S. (2016). Multicenter matrix-assisted laser desorption ionization-time of flight mass spectrometry study for identification of clinically relevant *Nocardia* spp. *J. Clin. Microbiol.* 54 1251–1258. 10.1128/JCM.02942-15 26912758 PMC4844739

[B9] Boratyn G. M. Camacho C. Cooper P. S. Coulouris G. Fong A. Ma N. (2013). BLAST: a more efficient report with usability improvements. *Nucleic Acids Res.* 41 W29–W33. 10.1093/nar/gkt282 23609542 PMC3692093

[B10] Buchman G. W. Banerjee S. Hansen J. N. (1988). Structure, expression, and evolution of a gene encoding the precursor of nisin, a small protein antibiotic. *J. Biol. Chem.* 263 16260–16266. 3141403

[B11] Cavera V. L. Arthur T. D. Kashtanov D. Chikindas M. L. (2015). Bacteriocins and their position in the next wave of conventional antibiotics. *Int. J. Antimicrob. Agents* 46 494–501. 10.1016/j.ijantimicag.2015.07.011 26341839

[B12] Chatterjee C. Paul M. Xie L. Van Der Donk W. A. (2005). Biosynthesis and mode of action of lantibiotics. *Chem. Rev.* 105 633–684. 10.1021/cr030105v 15700960

[B13] Chaumeil P. A. Mussig A. J. Hugenholtz P. Parks D. H. (2019). GTDB-Tk: a toolkit to classify genomes with the genome taxonomy database. *Bioinformatics* 15:btz848. 10.1093/bioinformatics/btz848 31730192 PMC7703759

[B14] Chen P. Qi F. Novak J. Caufield P. W. (1999). The specific genes for lantibiotic mutacin II biosynthesis in *Streptococcus mutans* T8 are clustered and can be transferred en bloc. *Appl. Environ. Microbiol.* 65 1356–1360. 10.1128/AEM.65.3.1356-1360.1999 10049909 PMC91190

[B15] Clarridge J. E. (2004). Impact of 16S rRNA gene sequence analysis for identification of bacteria on clinical microbiology and infectious diseases. *Clin. Microbiol. Rev.* 17 840–862. 10.1128/CMR.17.4.840-862.2004 15489351 PMC523561

[B16] Cleveland J. Montville T. J. Nes I. F. Chikindas M. L. (2001). Bacteriocins: safe, natural antimicrobials for food preservation. *Int. J. Food Microbiol.* 71 1–20. 10.1016/s0168-1605(01)00560-8 11764886

[B17] Collins F. W. J. Mesa-Pereira B. O’Connor P. M. Rea M. C. Hill C. Ross R. P. (2018). Reincarnation of bacteriocins from the *Lactobacillus* pangenomic graveyard. *Front. Microbiol.* 9:1298. 10.3389/fmicb.2018.01298 30013519 PMC6036575

[B19] Cotter P. D. Hill C. Ross R. P. (2005). Bacterial lantibiotics: strategies to improve therapeutic potential. *Curr. Protein Pept. Sci.* 6 61–75. 10.2174/1389203053027584 15638769

[B20] Cotter P. D. Ross R. P. Hill C. (2013). Bacteriocins-a viable alternative to antibiotics? *Nat. Rev. Microbiol.* 11 95–105. 10.1038/nrmicro2937 23268227

[B21] Delves-Broughton J. (1990). Nisin and its application as a food preservative. *Int. J. Dairy Technol.* 43 73–76. 10.1111/j.1471-0307.1990.tb02449

[B22] Diep D. B. Håvarstein L. S. Nissen-Meyer J. Nes I. F. (1994). The gene encoding plantaricin A, a bacteriocin from *Lactobacillus plantarum* C11, is located on the same transcription unit as an *agr*-like regulatory system. *Appl. Environ. Microbiol.* 60 160–166. 10.1128/aem.60.1.160-166.1994 8117074 PMC201284

[B23] Doyle M. P. Erickson M. C. Alali W. Cannon J. Deng X. Ortega Y. (2015). The food industry’s current and future role in preventing microbial foodborne illness within the United States. *Clin. Infect. Dis.* 61 252–259. 10.1093/cid/civ253 25824814

[B24] Eddy S. R. (2011). Accelerated profile HMM searches. *PLoS Comput. Biol.* 7:e1002195. 10.1371/journal.pcbi.1002195 22039361 PMC3197634

[B25] Field D. Cotter P. D. Hill C. Ross R. P. (2015). Bioengineering lantibiotics for therapeutic success. *Front. Microbiol.* 6:1363. 10.3389/fmicb.2015.01363 26640466 PMC4662063

[B26] Ghyselinck J. Van Hoorde K. Hoste B. Heylen K. De Vos P. (2011). Evaluation of MALDI-TOF MS as a tool for high-throughput dereplication. *J. Microbiol. Methods* 86 327–336. 10.1016/j.mimet.2011.06.004 21699925

[B27] Giraffa G. Chanishvili N. Widyastuti Y. (2010). Importance of lactobacilli in food and feed biotechnology. *Res. Microbiol.* 161 480–487. 10.1016/j.resmic.2010.03.001 20302928

[B28] Guo Y. Huang E. Yuan C. Zhang L. Yousef A. E. (2012). Isolation of a *Paenibacillus* sp. strain and structural elucidation of its broad-spectrum lipopeptide antibiotic. *Appl. Environ. Microbiol.* 78 3156–3165. 10.1128/AEM.07782-11 22367082 PMC3346447

[B29] He Z. Kisla D. Zhang L. Yuan C. Green-Church K. B. Yousef A. E. (2007). Isolation and identification of a *Paenibacillus polymyxa* strain that coproduces a novel lantibiotic and polymyxin. *Appl. Environ. Microbiol.* 73 168–178. 10.1128/AEM.02023-06 17071789 PMC1797129

[B30] Hillman J. D. Novák J. Sagura E. Gutierrez J. A. Crowley P. J. Hess M. (1998). Genetic and biochemical analysis of mutacin 1140, a lantibiotic from *Streptococcus mutans*. *Inf. Imm.* 66 2743–2749. 10.1128/iai.66.6.2743-2749.1998 PMC108264 9596742

[B31] Huang C. H. Lee F. L. Liou J. S. (2010). Rapid discrimination and classification of the Lactobacillus plantarum group based on a partial dnaK sequence and DNA fingerprinting techniques. *Antonie Van Leeuwenhoek Int. J. Gen. Mol. Microbiol.* 97 289–296. 10.1007/s10482-009-9409-5 20039128

[B32] Huang C. H. Li S. W. Huang L. Watanabe K. (2018). Identification and classification for the *Lactobacillus casei* group. *Front. Microbiol.* 9:1974. 10.3389/fmicb.2018.01974 30186277 PMC6113361

[B33] Hussein W. E. Huang E. Ozturk I. Yousef A. E. (2019). Draft genome sequence of *Lactobacillus paraplantarum* OSY-TC318, a producer of the novel lantibiotic paraplantaracin TC318. *Microbiol. Resour. Announc.* 8:19. 10.1128/mra.00274-19 31072901 PMC6509526

[B34] Huys G. Vancanneyt M. D’Haene K. Vankerckhoven V. Goossens H. Swings J. (2006). Accuracy of species identity of commercial bacterial cultures intended for probiotic or nutritional use. *Res. Microbiol.* 157 803–810. 10.1016/j.resmic.2006.06.006 16919915

[B35] Hyatt D. Chen G. L. LoCascio P. F. Land M. L. Larimer F. W. Hauser L. J. (2010). Prodigal: prokaryotic gene recognition and translation initiation site identification. *BMC Bioinformatics* 11:119. 10.1186/1471-2105-11-119 20211023 PMC2848648

[B36] Jack R. Götz F. Jung G. (2008). “Lantibiotics,” in *Biotechnology, Volume 7: Products of Secondary Metabolism*, 2nd Edn, eds Rehm H. J. Reed G. (Weinheim: Wiley), 323–368. 10.1002/9783527620890.ch8

[B37] Jain C. Rodriguez-R L. M. Phillippy A. M. Konstantinidis K. T. Aluru S. (2018). High throughput ANI analysis of 90K prokaryotic genomes reveals clear species boundaries. *Nat. Commun.* 9:5114. 10.1038/s41467-018-07641-9 30504855 PMC6269478

[B38] Kellner R. Jung G. Hörner T. Zähner H. Schnell N. Entian K. D. (1988). Gallidermin: a new lanthionine-containing polypeptide antibiotic. *Eur. J. Biochem.* 177 53–59. 10.1111/j.1432-1033.1988.tb14344.x-i2 3181159

[B39] Kleerebezem M. Boekhorst J. Van Kranenburg R. Molenaar D. Kuipers O. P. Leer R. (2003). Complete genome sequence of *Lactobacillus plantarum* WCFS1. *Proc. Natl. Acad. Sci. U.S.A.* 100 1990–1995. 10.1073/pnas.0337704100 12566566 PMC149946

[B40] Knudsen G. M. Chalkley R. J. (2011). The effect of using an inappropriate protein database for proteomic data analysis. *PLoS One* 6:e20873. 10.1371/journal.pone.0020873 21695130 PMC3114852

[B41] Letunic I. Bork P. (2019). Interactive tree of life (iTOL) v4: recent updates and new developments. *Nucleic Acids Res.* 47 W256–W259. 10.1093/nar/gkz239 30931475 PMC6602468

[B42] Maldonado A. Jiménez-Díaz R. Ruiz-Barba J. L. (2004). Induction of plantaricin production in *Lactobacillus plantarum* NC8 after coculture with specific Gram-positive bacteria is mediated by an autoinduction mechanism. *J. Bacteriol.* 186 1556–1564. 10.1128/JB.186.5.1556-1564.2004 14973042 PMC344433

[B43] Matsen F. A. Kodner R. B. Armbrust E. V. (2010). Pplacer: linear time maximum-likelihood and bayesian phylogenetic placement of sequences onto a fixed reference tree. *BMC Bioinform.* 11:538. 10.1186/1471-2105-11-538 21034504 PMC3098090

[B44] Mazzotta A. S. Crandall A. D. Montville T. J. (1997). Nisin resistance in *Clostridium botulinum* spores and vegetative cells. *Appl. Environ. Microbiol.* 63 2654–2659. 10.1128/aem.63.7.2654-2659.1997 16535641 PMC1389196

[B45] Mendler K. Chen H. Parks D. H. Lobb B. Hug L. A. Doxey A. C. (2019). AnnoTree: visualization and exploration of a functionally annotated microbial tree of life. *Nucleic Acids Res.* 47 4442–4448. 10.1093/nar/gkz246 31081040 PMC6511854

[B46] Ming X. Daeschel M. A. (1993). Nisin resistance of foodborne disease and the specific resistance responses of *Listeria monocytogenes* Scott A. *J. Food Prot.* 56 944–948. 10.4315/0362-028x-56.11.944 31113089

[B47] Mokoena M. P. (2017). Lactic acid bacteria and their bacteriocins: classification, biosynthesis and applications against uropathogens: a mini-review. *Molecules* 22:1255. 10.3390/molecules22081255 28933759 PMC6152299

[B48] Montalbán-López M. Zhou L. Buivydas A. van Heel A. J. Kuipers O. P. (2012). Increasing the success rate of lantibiotic drug discovery by synthetic biology. *Expert Opin. Drug Discov.* 7 695–709. 10.1517/17460441.2012.693476 22680308

[B49] Mota-Meira M. Lacroix C. LaPointe G. Lavoie M. C. (1997). Purification and structure of mutacin B-Ny266: a new lantibiotic produced by *Streptococcus mutans*. *FEBS Lett.* 410 275–279. 10.1016/S0014-5793(97)00425-0 9237644

[B50] Nes I. F. Johnsborg O. (2004). Exploration of antimicrobial potential in LAB by genomics. *Curr. Opin. Biotechnol.* 15 100–104. 10.1016/j.copbio.2004.02.001 15081046

[B51] Parks D. H. Chuvochina M. Chaumeil P. A. Rinke C. Mussig A. J. Hugenholtz P. (2019). Selection of representative genomes for 24,706 bacterial and archaeal species clusters provide a complete genome-based taxonomy. *bioRxiv* [Preprint], 10.1101/771964

[B52] Parks D. H. Chuvochina M. Waite D. W. Rinke C. Skarshewski A. Chaumeil P. A. (2018). A standardized bacterial taxonomy based on genome phylogeny substantially revises the tree of life. *Nat. Biotechnol.* 36:996. 10.1038/nbt.4229 30148503

[B53] Perez R. H. Zendo T. Sonomoto K. (2014). Novel bacteriocins from lactic acid bacteria (LAB): various structures and applications. *Microb. Cell Fact* 13:S3. 10.1186/1475-2859-13-S1-S3 25186038 PMC4155820

[B54] Qi F. Chen P. Caufield P. W. (2000). Purification and biochemical characterization of mutacin I from the group I strain of *Streptococcus mutans*, CH43, and genetic analysis of mutacin I biosynthesis genes. *Appl. Environ. Microbiol.* 66 3221–3229. 10.1128/AEM.66.8.3221-3229.2000 10919773 PMC92137

[B55] Repka L. M. Chekan J. R. Nair S. K. Van Der Donk W. A. (2017). Mechanistic understanding of lanthipeptide biosynthetic enzymes. *Chem. Rev.* 117 5457–5520. 10.1021/acs.chemrev.6b00591 28135077 PMC5408752

[B56] Rhodehamel E. J. Harmon S. M. (2001). *Bacteriological Analytical Manual, Chapter 16, Clostridium Perfringens.* Washington, DC: Food and Drug Administrtaion.

[B57] Riley M. A. Wertz J. E. (2002). Bacteriocin diversity: ecological and evolutionary perspectives. *Biochimie* 84 357–364. 10.1016/S0300-9084(02)01421-9 12423779

[B58] Rojo-Bezares B. Sáenz Y. Navarro L. Jiménez-Díaz R. Zarazaga M. Ruiz-Larrea F. (2008). Characterization of a new organization of the plantaricin locus in the inducible bacteriocin-producing *Lactobacillus plantarum* J23 of grape must origin. *Arch. Microbiol.* 189 491–499. 10.1007/s00203-007-0342-6 18193201

[B59] Sahl H. G. Bierbaum G. (1998). Lantibiotics: biosynthesis and biological activities of uniquely modified peptides from Gram-positive bacteria. *Annu. Rev. Microbiol.* 52 41–79. 10.1146/annurev.micro.52.1.41 9891793

[B60] Sandiford S. K. (2015). Perspectives on lantibiotic discovery - where have we failed and what improvements are required? *Expert Opin. Drug Discov.* 10 315–320. 10.1517/17460441.2015.1016496 25697059

[B61] Sandiford S. K. (2017). Genome database mining for the discovery of novel lantibiotics. *Expert Opin. Drug Discov.* 12 489–495. 10.1080/17460441.2017.1303475 28306363

[B62] Sharma C. Rokana N. Chandra M. Singh B. P. Gulhane R. D. Gill J. P. S. (2018). Antimicrobial resistance: its surveillance, impact, and alternative management strategies in dairy animals. *Front. Vet. Sci.* 4:237. 10.3389/fvets.2017.00237 29359135 PMC5766636

[B63] Singh N. P. Tiwari A. Bansal A. Thakur S. Sharma G. Gabrani R. (2015). Genome level analysis of bacteriocins of lactic acid bacteria. *Comput. Biol. Chem.* 56 1–6. 10.1016/j.compbiolchem.2015.02.013 25733445

[B64] Smith L. Novaandk J. Rocca J. McClung S. Hillman J. D. Edison A. S. (2000). Covalent structure of mutacin 1140 and a novel method for the rapid identification of lantibiotics. *Eur. J. Biochem.* 267 6810–6816. 10.1046/j.1432-1033.2000.01777 11082191

[B65] Stiles M. E. (1996). Biopreservation by lactic acid bacteria. *Antonie Van Leeuwenhoek* 70 331–345. 10.1007/BF00395940 8879414

[B66] Stiles M. E. Holzapfel W. H. (1997). Lactic acid bacteria of foods and their current taxonomy. *Int. J. Food Microbiol.* 36 1–29. 10.1016/S0168-1605(96)01233-0 9168311

[B67] Torriani S. Clementi F. Vancanneyt M. Hoste B. Dellaglio F. Kersters K. (2001). Differentiation of *Lactobacillus plantarum*, *L. pentosus* and *L. paraplantarum* species by RAPD-PCR and AFLP. *Syst. Appl. Microbiol.* 24 554–560. 10.1078/0723-2020-00071 11876363

[B68] van der Donk W. A. Nair S. K. (2014). Structure and mechanism of lanthipeptide biosynthetic enzymes. *Curr. Opin. Struct. Biol.* 29 58–66. 10.1016/J.SBI.2014.09.006 25460269 PMC4267917

[B69] van Heel A. J. Montalban-Lopez M. Oliveau Q. Kuipers O. P. (2017). Genome-guided identification of novel head-to-tail cyclized antimicrobial peptides, exemplified by the discovery of pumilarin. *Microb. Genomics* 3:134. 10.1099/mgen.0.000134 29177092 PMC5695211

[B70] Verraes C. Van Boxstael S. Van Meervenne E. Van Coillie E. Butaye P. Catry B. (2013). Antimicrobial resistance in the food chain: a review. *Int. J. Environ. Res. Public Health* 10 2643–2669. 10.3390/ijerph10072643 23812024 PMC3734448

[B71] Walsh C. J. Guinane C. M. Hill C. Ross R. P. O’toole P. W. Cotter P. D. (2011). In silico identification of bacteriocin gene clusters in the gastrointestinal tract, based on the human microbiome project’s reference genome database. *BMC Microbiol.* 15:183. 10.1186/s12866-015-0515-4 26377179 PMC4573289

[B72] Weisburg W. G. Barns S. M. Pelletier D. A. Lane D. J. (1991). 16S ribosomal DNA amplification for phylogenetic study. *J. Bacteriol.* 173 697–703. 10.1128/jb.173.2.697-703.1991 1987160 PMC207061

[B73] Whitman W. B. (2016). Modest proposals to expand the type material for naming of prokaryotes. *Int. J. Syst. Evol. Microbiol.* 66 2108–2112. 10.1099/ijsem.0.000980 26902077

[B74] Willey J. M. van der Donk W. A. (2007). Lantibiotics: peptides of diverse structure and function. *Annu. Rev. Microbiol.* 61 477–501. 10.1146/annurev.micro.61.080706.093501 17506681

[B75] Yang X. Huang E. Yuan C. Zhang L. Yousef A. E. (2016). Isolation and structural elucidation of brevibacillin, an antimicrobial lipopeptide from *Brevibacillus laterosporus* that combats drug-resistant Gram-positive bacteria. *Appl. Environ. Microbiol.* 82 2763–2772. 10.1128/AEM.00315-16 26921428 PMC4836408

[B76] Zhu H. Sandiford S. K. Van Wezel G. P. (2014). Triggers and cues that activate antibiotic production by actinomycetes. *J. Ind. Microbiol. Biotechnol.* 41 371–386. 10.1007/s10295-013-1309-z 23907251

