# Supplementary material for: Genome-Guided Mass Spectrometry Expedited the Discovery of Paraplantaricin TC318, a Lantibiotic Produced by Lactobacillus paraplantarum Strain Isolated From Cheese
Source: Front Microbiol. 2020 Jul 14;11:1381. doi: 10.3389/fmicb.2020.01381 (PMC7372301; doi:10.3389/fmicb.2020.01381)
Supplement: Supplementary file 1 [file Table_1.docx]

Table S1. List of structurally characteristic fragment ions obtained by collision induced dissociation (80 eV) of the [M+H]^+^ protonated paraplantaricin TC318 peptide using MALDI^a^.

| **Ions^b^** | **Structure** | **Chemical Formula** | **Calculated *m/z*** | **Measured *m/z*** | **Relative error (ppm)** | **Corresponding neutral loss** |
| --- | --- | --- | --- | --- | --- | --- |
| **[M+H]^+^** | 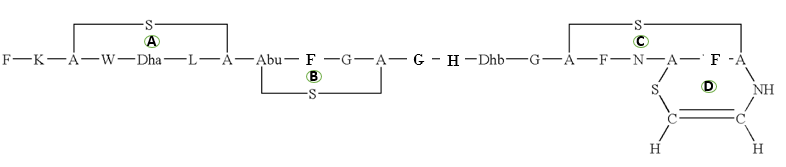 | C_106_H_134_N_27_O_22_S_4_ | 2264.90741 | 2264.90718 | 0.10 | N/A |
| 1 | 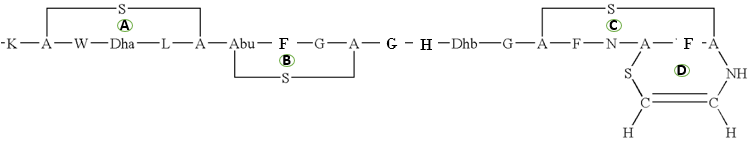 | C_97_H_125_N_26_O_21_S_4_ | 2117.83900 | 2117.83450 | 2.12 | -F |
| 2 | 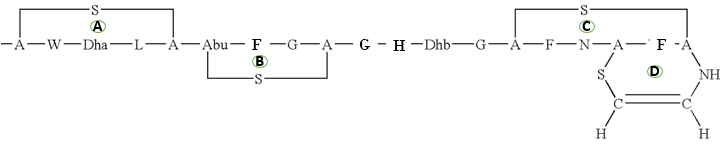 | C_91_H_113_N_24_O_20_S_4_ | 1989.74403 | 1989.73826 | 2.90 | -FK |
| 3 | 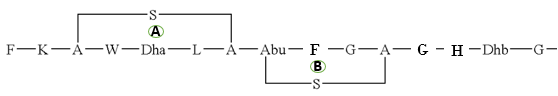 | C_73_H_94_N_19_O_15_S_2_ | 1540.66127 | 1540.65731 | 2.57 | -Ring C+D |
| 4 | 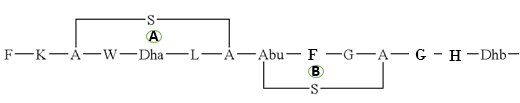 | C_71_H_91_N_18_O_14_S_2_ | 1483.63981 | 1483.63618 | 2.45 | 3-G |
| 5 | 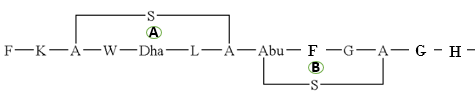 | C_67_H_86_N_17_O_13_S_2_ | 1400.60269 | 1400.59940 | 2.35 | 4-Dhb |
| 6 | 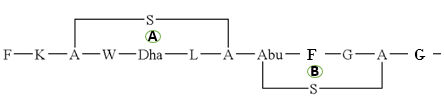 | C_61_H_79_N_14_O_12_S_2_ | 1263.54378 | 1263.54112 | 2.10 | 5-H |
| 7 | 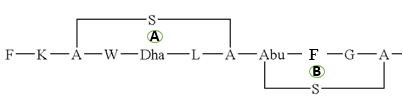 | C_59_H_76_N_13_O_11_S_2_ | 1206.52232 | 1206.51965 | 2.21 | 6-G |
| 8 | 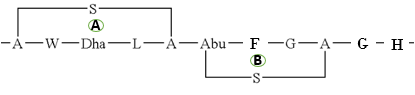 | C_52_H_65_N_14_O_11_S_2_ | 1125.43932 | 1125.43666 | 2.36 | 5-FK |
| 9 | 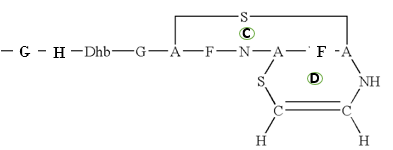 | C_47_H_59_N_14_O_11_S_2_ | 1059.39237 | 1059.39045 | 1.81 | 2-Ring A+B |

^a^ For simplicity, fragment ions associated with loss of ammonia and water and/or combination of these are not shown; monoisotopic m/z values of ions are shown. ^b^ Numbering in Figures S1a-e

1

Figure S1. Fragmentation of paraplantaricin TC318

S1a) 1: Mass of singly charged ion, 2264.90718 minus 2117.83450 corresponds to mass of phenylalanine (F)

2

S1b) 2: Mass of singly charged ion, 2264.90718 minus 1989.73826 corresponds to mass of phenylalanine (F)+lysine (K)

G

H

Dhb

G

6

1206.51965

7

5

4

3

S1c) 3: Mass of singly charged ion, 2264.90718 minus 1540.65731 corresponds to mass of ring (C) + ring (D)

4: 1540.65731 minus 1483.63618 corresponds to mass of glycine (G)

5: 1483.63618 minus 1400.59940 corresponds to mass of didehydrobutyric acid (Dhb)

6: 1400.59940 minus 1263.54112 corresponds to mass of histidine (H)

7: 1263.54112 minus 1206.51965 corresponds to mass of glycine (G)

S1d) 8: 2264.90718 minus 1125.43666 corresponds to peak 5 minus mass of phenylalanine (F) + lysine (K)

8

9

S1e) 9: Peak 9 corresponds to peak 2 minus mass of ring (A) + ring (B)


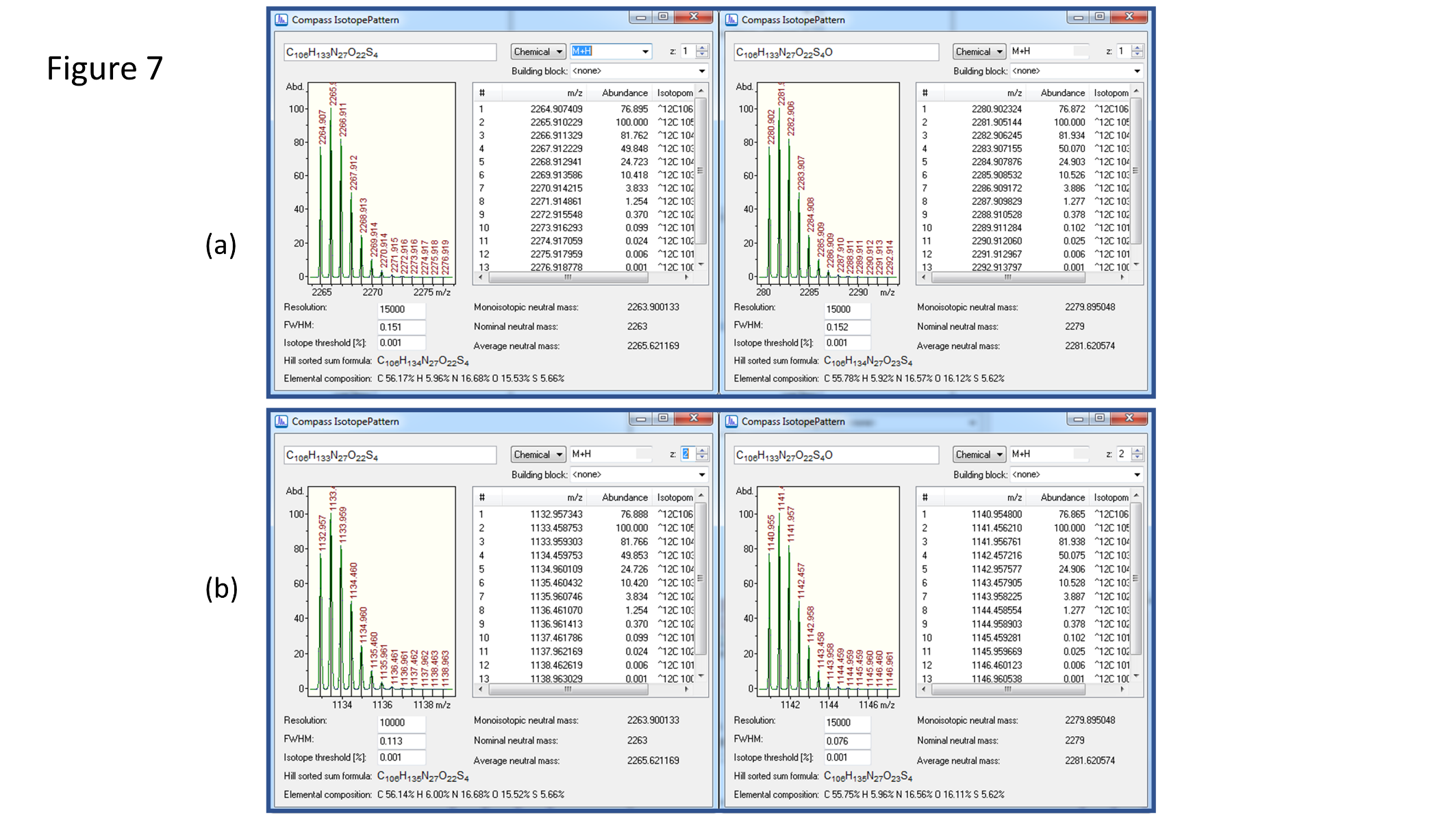


Figure S2. Theoretical molecular masses of paraplantaricin TC318 (a) singly charged ions and (b) doubly charged ion with the corresponding chemical formulas using [Protein Prospector](http://prospector.ucsf.edu/) v5.22.0.
